# Supplementary material for: Variables Determining Higher Home Care Effectiveness in Patients with Chronic Cardiovascular Disease
Source: Int J Environ Res Public Health. 2022 Apr 24;19(9):5170. doi: 10.3390/ijerph19095170 (PMC9102908; doi:10.3390/ijerph19095170)
Supplement: Supplementary file 1 [file ijerph-19-05170-s001.zip › ijerph-1644166-supplementary.pdf]

Suppl. Table S1. Sociodemographic data of CVD patients with LEHC (n = 84*) and HEHC (n = 85*).

Variable		↓ LEHC				↑ HEHC				Fisher test - p	
		**n**	**%**			**n**	**%**				
**Sex**	women	54	65.1			62	72.9			0.28	
	men	29	34.9			23	27.1				
		**n**	**Me**	**q1**	**q3**	**n**	**Me**	**q1**	**q3**	**Wilcoxon test**	
Age (in years)		84	77.5	64.8	85	85	70	56	82	**W**	**p**
										4254	0.032
Duration of CVD illness (in years)		81	10			83	10			3471.5	0.718
**Variable**	**Categories**	**n**	**%**			**n**	**%**			**Fisher test - p**	
Education	primary	34	40.5			19	22.4			0.025	
	vocational	22	26.2			22	25.9				
	secondary without Matura Exam	18	21.4			23	27.1				
	secondary with Matura Exam	4	4.8			5	5.9				
	post-secondary	2	2.4			2	2.4				
	higher BA/MA	4	4.8			14	16.5				
	total	84	100			85	100				
Staying in a relationship	no	45	54.2			32	38.6			0.085	
	yes	38	45.8			51	61.4				
	total	83	100			83	100				
Place of residence	urban	49	58.3			47	55.3			0.757	
	rural	35	41.7			38	44.7				
	total	84	100			85	100				
Financial status	very good (above PLN 3001 per person in the family)	1	1.2			4	4.9			0.004	
	good (from PLN 2001-3000 per person in family)	13	15.9			28	34.6				
	average (from PLN 1001-2000 per person in family)	47	57.3			41	50.6				
	bad (from PLN 501-1000 per person in family)	21	25.6			8	9.9				
	total	82	100			81	100				
Social benefit (data from a nurse)	yes	18	21.4			6	7.2			0.007	
	no	66	78.6			77	92.8				
	total	84	100			83	100				

Legend: LEHC - patients with worse effectiveness of medical care; HEHC - patients with better effectiveness of medical care; BA - bachelor's degree; MA - master's degree; n - group size; % - percentage; Me - median; q1 and q3 – first and third quartiles; W - Wilcoxon test - p ≤ 0.05; Fischer test - p ≤ 0.05. * Numbers in column n do not sum to 84 and 85 due to missing data.

Supplementary Figures S1 and S2 show the distributions of the quantitative variables listed in Suppl. Table S1 and Table 1. The box-and-whisker plot (described in the lower right corner of Figure S1) was used for this purpose. The difference between the upper quartile and the lower quartile is the interquartile range (IQR). The lower whisker is drawn from the lower end of the box to the smallest value that is no smaller ten 1.5 IQR below the lower quartile. Similarly, the upper whisker is drawn from the middle of the upper end of the box to largest value that is no larger then 1.5 IQR above the upper quartile. (The rationale for these definition is that when data are drawn from the normal distribution or other distribiution with a similar shape, about 99% of the observations wil fall between the whiskers).

Suppl. Figure S1. The distributions of the quantitative variables listed in Suppl. Table S1 and Table 1 (part 1).

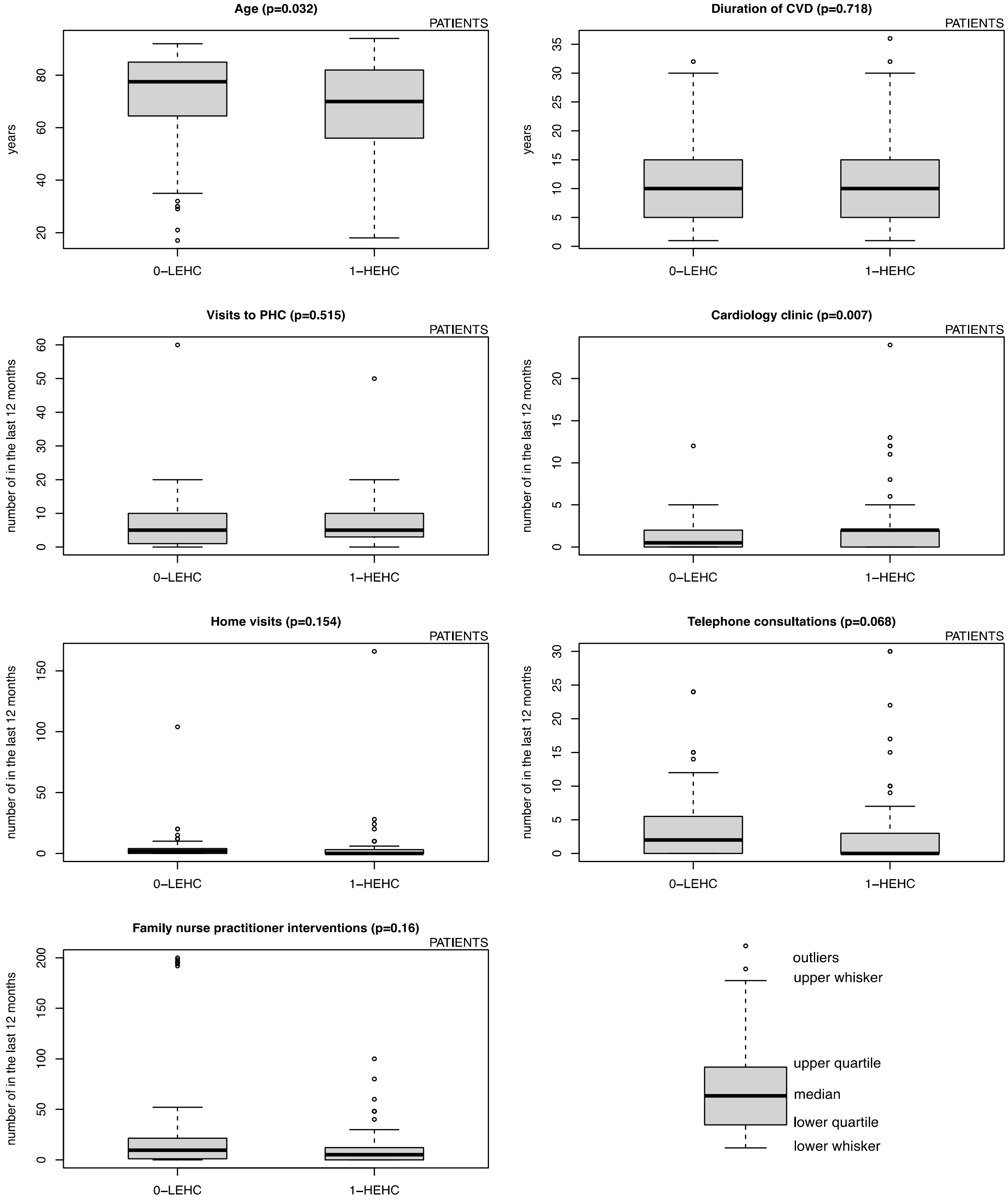

Suppl. Figure S2. The distributions of the quantitative variables listed in Table 1 (part 2).

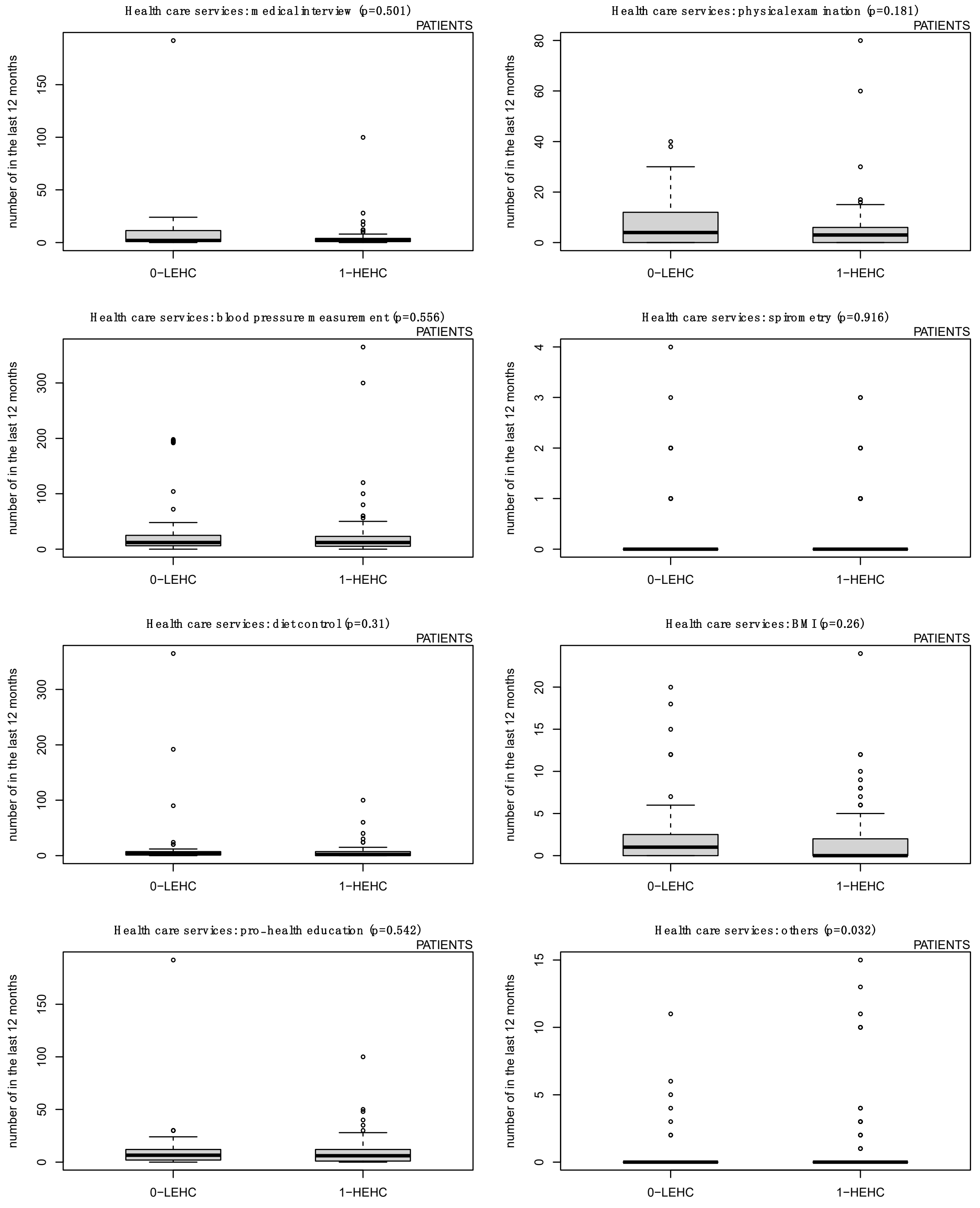

Suppl. Table S2. Differences between caregivers of patients with LEHC (n = 77) and HEHC (n = 57) - by 25% quantile.

**Variable**		**↓ CP with LEHC**				**↑ CP with HEHC**				**Fisher test -p**	
		**n**	**%**			**n**	**%**				
**Sex**	women	54	70.1			35	61.4			0.356	
	men	23	29.9			22	38.6				
Education	primary	10	13			2	3.5			0.258	
	vocational	16	20.8			16	28.1				
	secondary without Matura Exam	4	5.2			1	1.8				
	secondary with Matura Exam	21	27.3			11	19.3				
	post-secondary	5	6.5			5	8.8				
	BA	3	3.9			2	3.5				
	MA	18	23.4			20	35.1				
	total	77	100			57	100				
		**n**	**Me**	**q1**	**q3**	**n**	**Me**	**q1**	**q3**	**Wilcoxon test**	
										**W**	**p**
Age (in years)		76	54	43.75	62.5	56	52.5	42	63.5	2181.5	0.807
Period of homecare (in years)		61	4	2	7	47	4	2	10.5	1371.5	0.701
Camberwell Index		77	0.82	0.72	0.94	57	0.88	0.79	0.94	1908.5	0.198
WHOQOL-BREFF	Qol perception	77	1	1	2	57	1	1	2	2331.5	0.491
	health perception	77	4	3	5	57	4	2	5	2326	0.528
	physical domain	77	13.14	10.86	16	57	14.29	12	17.14	1726	0.035
	psychological domain	77	12.67	11.33	14	56	14	12.67	15.33	1499	0.003
	social relations domain	77	14.67	12	16	57	16	13.33	17.33	1754.5	0.044
	environmental domain	77	12	11	12.5	57	12	11.5	13	1909.5	0.197
HBI	sum	73	80	72	92	54	87	73	96.75	1599.5	0.07
	proper eating habits	75	3.33	2.67	3.83	54	3.58	2.83	4.17	1754	0.196
	preventive behaviours	75	3.67	3.17	4.17	54	3.83	3.21	4.17	1868	0.454
	proper mental attitudes	74	3.5	3	4	54	3.75	3.33	4.17	1565.5	0.036
	health practices	74	3.17	2.5	3.67	54	3.5	2.83	3.96	1646.5	0.09
HADS-M	anxiety	76	12	10	13	54	11	10	13	2071	0.93
	depression	70	12	11	13	51	12	11	13	1947.5	0.386
	aggression	76	3	2	4	56	4	2	5	1708.5	0.048

Legend: CP with LEHC - caregivers of patients with worse health care effectiveness; CP with HEHC - caregivers of patients with better health care effectiveness; n - group size; % - percentage; Me - Median; q1 and q3 – first and third quartiles; BA - bachelor's degree; MA - master's degree; W - Wilcoxon test - $p \leq 0.05$; Fischer test - $p \leq 0.05$. * Numbers in column n do not sum to 77 and 57 due to missing data.

Suppl. Figure S3. The distributions of the quantitative variables listed in Suppl. Table S2 (part 1).

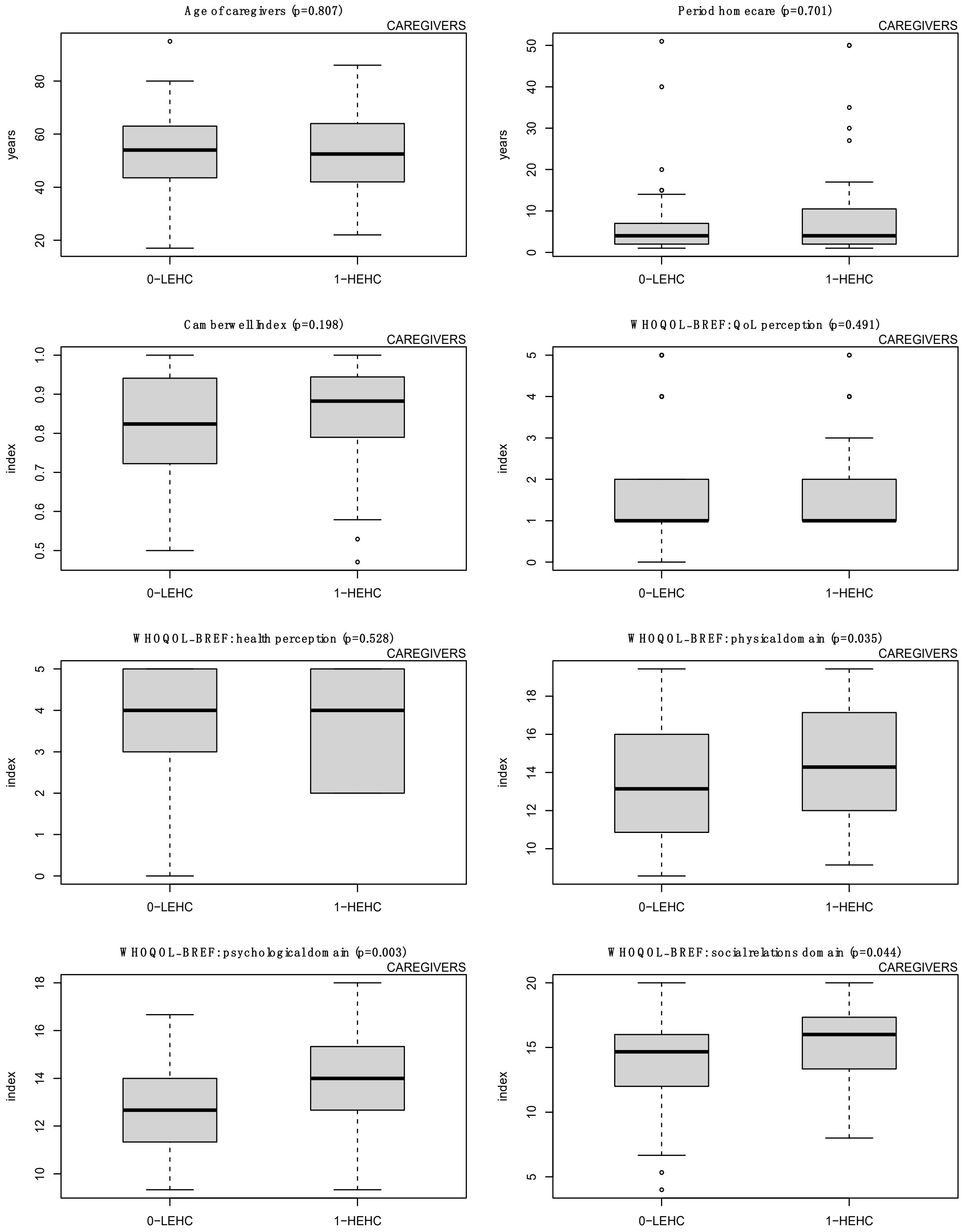

Suppl. Figure S4. The distributions of the quantitative variables listed in Suppl. Table S2 (part 2).

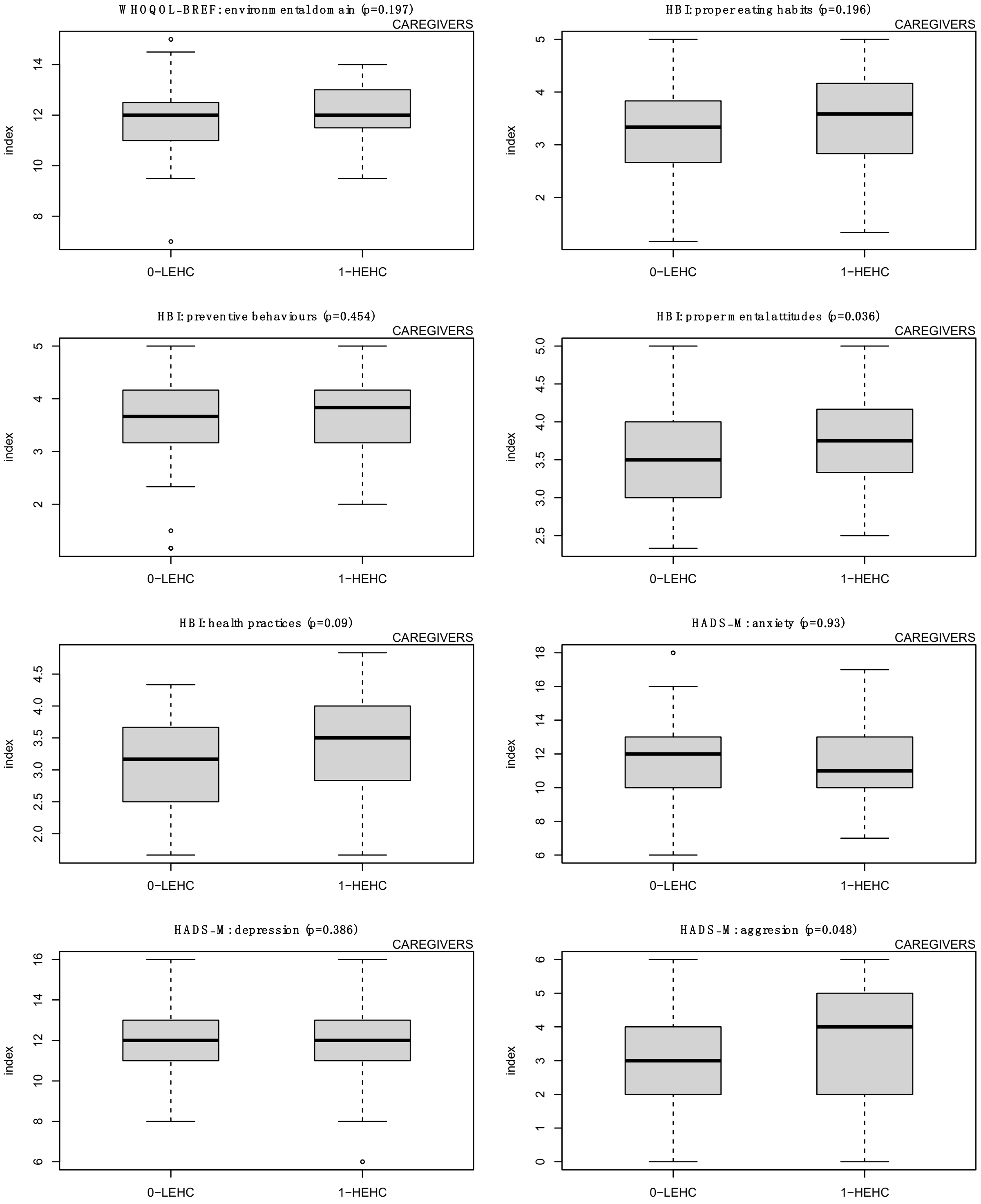

Supplementary results logistic regression analysis and odds ratio in Table 2

Logistic regression analysis and odds ratio – model 1 (n = 130)

Patients who regularly take prescribed medications have a 4.08 (OR = 0.25, 95% CI: 0.07-0.72)-fold higher chance of better EHC than patients who do not.

Patients who have a positive attitude toward the disease have a 5.45 (OR = 0.18, 95% CI: 0.06-0.48)-fold higher chance of better EHC than patients with a negative attitude.

Patients who have concomitant endocrine disorders have a 6.18 (OR = 0.16, 95% CI: 1.83-23.78)-fold higher risk of better EHC than patients who do not have such disorders.

Those patients whose caregivers report that they are full of hope and vigor after a disease visit have a 4.48 (OR = 4.48, 95% CI: 1.24–18.05) times greater chance of better EHC than those patients whose caregivers do not report such an attitude (Table 2).

Logistic regression analysis and odds ratio – model 2 (n = 130)

Patients who used 15 health care services in the past 12 months were 29.60 (OR = 29.60, 95% CI: 1.48-1597.17) times more likely to have better EHC than patients who did not use any services in the same period. However, those with the number of services differing by 1 in their favor have 1.25 (OR = 1.25, 95% CI: 1.03-1.64) times higher odds of better EHC than those with a lower number (Table 2).

Logistic regression analysis and odds ratio – model 3 (n = 120)

The patients who do not have I99 disease diagnosis (according to ICD-10) have 6.42 (OR = 0.16, 95% CI: 0.02-0.90) times higher odds of better EHC than the patients with this disease.

Those patients whose caregivers report high HBI scores (10 on the HBI-Sten scale) have 68.94 (OR = 68.94, 95% CI: 1.70-4648.61) times greater odds of better EHC than those whose scores are low (1 on the HBI-Sten scale). However, those whose caregivers differ by 1 point in their favor on this score have 1.60 (OR = 1.60, 95% CI: 1.06-2.56) times greater odds.

Patients whose caregivers report low scores for positive mental attitude (2.33 on the HBI scale for positive mental attitude) have 58.5 (OR = 0.02, 95% CI: 0.00-0.81) times greater odds of better EHC than those for whom this score is high (5 on the HBI scale for positive mental attitude). However, when caregivers differ by 1 point on the scale, caregivers with a lower score are 4.60 (OR = 0.22, 95% CI: 0.04-0.92) times more likely to have better EHC than those with a higher score (Table 2).

Logistic regression analysis and odds ratio – model 4 (n = 120)

Those patients whose caregivers express higher expectations for the family caregiver's manual skills have a 4.08 (OR = 4.08, 95% CI: 1.31-14.33)-fold higher odds of better EHC than those whose caregivers do not express such expectations.

Those patients whose caregivers report high scores in the physical quality of life domain (19.43 on the WHOQOL-BREF scale) have a 10.09 (OR = 10.09, 95% CI: 1.66-76.09)-fold higher chance of better EHC than those whose caregivers have low scores in this domain (8.57 on the WHOQOL-BREF scale). However, those whose caregivers differ by 1 point in their favor in this regard have 1.24 (OR = 1.24, 95% CI: 1.05-1.49) times higher odds (Table 2).

Logistic regression analysis and odds ratio – model 5 (n = 124)

Those patients diagnosed with an endocrinologic condition (reported by a nurse) have a 3.46 (OR = 3.46, 95% CI: 1.08-12.35) times greater chance of better EHC than those who do not report it (Table 2).

Logistic regression analysis and odds ratio – model 6 (n = 126)

Those patients who rated their physical well-being as very good had 9.24 (OR = 9.24, 95% CI: 1.59-63.30) times higher odds of better EHC than those who reported very low physical well-being

scores. Those who differed by 1 point (on a 5-point scale) in their favor in this regard had 1.74 (OR = 1.74, 95% CI: 1.12-2.82) times higher odds (Table 2).
